# A Deep Neural Network Model using Random Forest to Extract Feature Representation for Gene Expression Data Classification

**DOI:** 10.1038/s41598-018-34833-6

**Published:** 2018-11-07

**Authors:** Yunchuan Kong, Tianwei Yu

**Affiliations:** Department of Biostatistics and Bioinformatics, Emory University, 1518 Clifton Rd, Atlanta, GA 30322 USA

## Abstract

In predictive model development, gene expression data is associated with the unique challenge that the number of samples (n) is much smaller than the amount of features (*p*). This “*n* ≪ *p*” property has prevented classification of gene expression data from deep learning techniques, which have been proved powerful under “*n* > *p*” scenarios in other application fields, such as image classification. Further, the sparsity of effective features with unknown correlation structures in gene expression profiles brings more challenges for classification tasks. To tackle these problems, we propose a newly developed classifier named Forest Deep Neural Network (fDNN), to integrate the deep neural network architecture with a supervised forest feature detector. Using this built-in feature detector, the method is able to learn sparse feature representations and feed the representations into a neural network to mitigate the overfitting problem. Simulation experiments and real data analyses using two RNA-seq expression datasets are conducted to evaluate fDNN’s capability. The method is demonstrated a useful addition to current predictive models with better classification performance and more meaningful selected features compared to ordinary random forests and deep neural networks.

## Introduction

In the field of bioinformatics, the development of computational methods for predicting clinical outcomes using profiling datasets with a large amount of variables has drawn great interest. In such datasets, the sample sizes tend to be very small compared to the number of predictors (genes), hence resulting in the $n\ll p$ issue. Moreover, existence of complex unknown correlation structures among predictors has brought more difficulty in prediction and feature extraction. Therefore, the prediction task has been formulated as a classification problem combined with feature representations, and related work tried to solve the problem by utilizing machine learning approaches such as random forests^1,2^, neural networks^3^, sparse linear models^4,5^ and support vector machines^6^. While the primary goal of these methods are to achieve high classification accuracy, efforts have also been put into learning effective feature representations. Literature shows that among the machine learning techniques, random forests^7^ (RF) have been an excellent tool to learn feature representations^8,9^, given their robust classification power and easily interpretable learning mechanism. This can be useful in building robust predictive models especially when the underlying structures in the feature space are complex and unknown.

Classification methods have been developed considering known functional links between features. For example, a variant of the Random Forest method has been proposed where the feature sub-sampling was conducted according to spatial information of genes on a known functional network^10^.Objective functions of the support vector machine and the logistic regression were modified by adding relational penalty terms, again based on known functional information^11–13^. Very recently, a method embedding protein-protein interaction feature graph directly into the deep neural network structure has also been proposed^14^. The authors of these methods have demonstrated that incorporating feature relation structures results in better classification performance. However, considering the functional relation structures explicitly requires external information in addition to gene expression values. This requirement cannot always be satisfied as the functional structure can be unknown or incomplete.

Trying to develop a powerful classifier which can implicitly extract sparse feature relations from an extremely large feature space, we intend to incorporate a forest “feature detector” with deep neural networks (DNN), which is one of the state-of-the-art learning techniques^15^. Although in recent years, deep learning models have been proved to be powerful tools in classification, their application in bioinformatics is limited due to the $n\ll p$ issue^16^. This is because cell populations and clinical subject populations exhibit large heterogeneity and data characteristics across various laboratories are inconsistent, resulting in gene expression datasets to have limited numbers of samples compared to the large numbers of features. On the other hand, deep learning usually requires a large amount of training samples such as in image classification^17^, therefore the contradiction obstructs the use of deep learning techniques in the field of bioinformatics. Based on these facts, modified deep learning models suitable for disease outcome classification using gene expression data with $n\ll p$ are in need.

Building a supervised feature detector on top of DNN classifiers is a natural choice to achieve sparse learning with less parameters compared to the usual DNN, for the following reasons: (1) the detector detects effective features in a supervised manner, i.e. using the information of training outcomes, resulting in accurate feature representations; (2) the input of the downstream DNN, which is the output of the feature detector, has a much smaller dimension compared to the original feature sets. Also, the rationale of employing random forests over other models lies in two aspects: (1) as an ensemble model, RF is able to output prediction results from all its base learners rather than a single predicted probability score; (2) the importance of features in each base learner can be easily obtained. The first aspect allows us to build downstream DNN following the feature detector, which cannot be achieved if the detector only outputs a single prediction such as in support vector machines and logistic regressions. The second aspect facilitates feature evaluation process for the entire integrated model, while other classifiers such as kernel based methods may not naturally embrace feature selection mechanism. To the best of our knowledge, no work has been done along this track for gene expression data. In the field of traditional machine learning research such as computer vision, the idea of stacking classifiers^18^ has been implemented and is now very popular in Kaggle data science competitions (https://www.kaggle.com). Nevertheless, stacking methods are mainly intended to cross-validate a large amount of multi-level models, and consequently require much larger number of instances (samples) than the number of features with no exception. In contrast, our new fDNN classifier with supervised forest feature detector is developed for $n\ll p$ sparse learning. In this paper, we justify our approach by demonstrating the classification performance on both synthetic data and real RNA-seq datasets.

## Methods and Materials

### Forest deep neural networks

Our newly proposed forest deep neural network (fDNN) model consists of two parts. The forest part serves as a feature detector to learn sparse representations from raw inputs with the supervision of training outcomes, and the DNN part serves as a learner to predict outcomes with the new feature representations. In the forest part, independent decision trees^19^ are constructed, and the forest is then an ensemble of the trees. Therefore, a natural choice of building the forest is the Random Forest model^7^. Other forest constructions are also possible. For example, one can use the network-guided forests^10^ if the feature space is structured and known, or the forest can be simply built through bagging trees^20^. In this paper, we only employ random forests as the feature detector.

In the fDNN model, a forest ${\mathcal F}$ is a collection of decision trees

$$ {\mathcal F} ({\rm{\Theta }})=\{{{\mathscr{J}}}_{m}({{\rm{\Theta }}}_{m})\},\,m=1,\ldots ,M,$$

where *M* is the total number of trees in the forest, Θ = {Θ_1_, …, Θ_*M*_} represents the parameters in ${\mathcal F}$. In random forests, Θ includes splitting variables and their splitting values. In the feature detection stage, ${\mathcal F}$ is fitted by the training data **X** and **y**, where ${\bf{X}}\in {{ {\mathcal R} }}^{n\times p}$ is the input data matrix with *n* samples and *p* features and ${\bf{y}}\in {{ {\mathcal R} }}^{n}$ is the outcome vector containing classification labels. Through the fitted forest, for any observation **x**_*i*_, *i* = 1, …, *n*, we obtain the prediction from each tree in ${\mathcal F}$:

$$f({{\bf{x}}}_{i};{\rm{\Theta }})={({T}_{1}({{\bf{x}}}_{i};{{\rm{\Theta }}}_{1}),\ldots ,{T}_{M}({{\bf{x}}}_{i};{{\rm{\Theta }}}_{M}))}^{T},$$

where ${T}_{m}({{\bf{x}}}_{i};{{\rm{\Theta }}}_{m})={\hat{y}}_{im}$ is the binary prediction of observation **x**_*i*_ given by ${{\mathscr{J}}}_{m}$. Hence, denote **f**_*i*_: = *f*(**x**_*i*_;Θ) for simplicity, for an observation **x**_*i*_, **f**_*i*_ is a binary vector summarizing the signal detected from the forest and later on serves as the new input features to be fed into the DNN.

Following the new feature representations provided by the forest, the deep neural network with *l* hidden layers has a standard architecture

$$\begin{array}{rll}Pr({\bf{y}}|{\bf{F}},{\rm{\Psi }}) & = & g({{\bf{Z}}}_{out}{{\bf{W}}}_{out}+{{\bf{b}}}_{out})\\ {{\bf{Z}}}_{out} & = & \sigma ({{\bf{Z}}}_{l}{{\bf{W}}}_{l}+{{\bf{b}}}_{l})\\ \ldots \\ {{\bf{Z}}}_{k+1} & = & \sigma ({{\bf{Z}}}_{k}{{\bf{W}}}_{k}+{{\bf{b}}}_{k})\\ \ldots \\ {{\bf{Z}}}_{1} & = & \sigma ({\bf{F}}{{\bf{W}}}_{{in}}+{{\bf{b}}}_{{in}}),\end{array}$$

where **F** = (**f**_*i*_, …, **f**_*M*_)^*T*^ is the forest matrix with *n* samples and *M* tree predictions, **y** again is the classification outcome vector, Ψ denotes all the parameters in the DNN model, **Z**_*out*_ and **Z**_*k*_, *k* = 1, …, *l* − 1 are hidden neurons with corresponding weight matrices **W**_*out*_, **W**_*k*_ and bias vectors **b**_*out*_, **b**_*k*_. The dimensions of **Z** and **W** depend on the number of hidden neurons *h*_*in*_ and *h*_*k*_, *k* = 1, …, *l*, as well as the input dimension *M* and the number of classes *h*_*out*_. For binary classification problems, *h*_*out*_ ≡ 2 since the elements of ***y*** are binary. Usually, the number of hidden neurons decreases from the input layer, namely *h*_*in*_ = *M* > *h*_1_ > *h*_2_ … > *h*_*out*_. *σ*(⋅) is the activation function such as sigmoid, hyperbolic tangent or rectifiers. *g*(⋅) is the softmax function converting values of the output layer into probability prediction i.e.

$${p}_{i}=g({\mu }_{i1})=\frac{{e}^{{\mu }_{i1}}}{{e}^{{\mu }_{i0}}+{e}^{{\mu }_{i1}}}$$

where

$$\begin{array}{lll}{p}_{i} & := & Pr({y}_{i}=\mathrm{1|}{{\bf{f}}}_{i})\\ {\mu }_{i0} & := & {[{{\bf{z}}}_{i}^{(out)}]}^{T}{{\bf{w}}}_{0}^{(out)}+{{\bf{b}}}_{i}^{(out)}\\ {\mu }_{i1} & := & {[{{\bf{z}}}_{i}^{(out)}]}^{T}{{\bf{w}}}_{1}^{(out)}+{{\bf{b}}}_{i}^{(out)},\end{array}$$

where *i* = 1, …, *n*.

**Figure 1:**
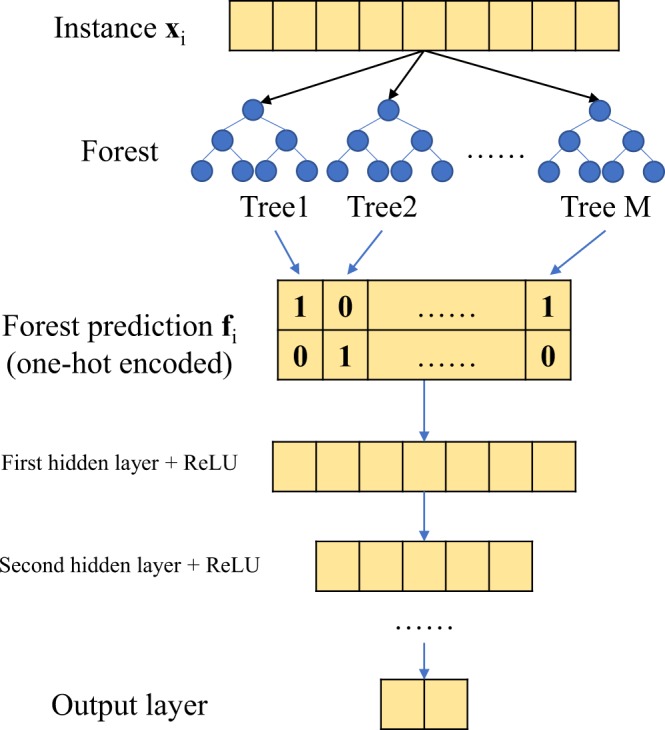
Visualization of the architecture of the fDNN model.

The parameters to be estimated in the DNN are thus all the weights and biases. The model can be trained using a stochastic gradient decent (SGD) based algorithm^21^ by minimizing the cross-entropy loss function

$$ {\mathcal L} ({\rm{\Psi }})=-\,\frac{1}{n}\sum _{i=1}^{n}\,\{{y}_{i}log\,({\hat{p}}_{i})+\mathrm{(1}-{y}_{i})\,log\,\mathrm{(1}-{\hat{p}}_{i})\},$$

where again Ψ denotes all the model parameters, and ${\hat{p}}_{i}$ is the fitted value of *p*_*i*_. More details about DNN can be found in standard deep learning reviews^21^. The entire architecture of the fDNN model is visualized in Fig. 1.

### Details of model training

The training of fDNN classifier consists of two stages. In the first stage, training data including labels are used to fit the forest, and predictions from each tree in the forest for all instances are then fed into the fully-connected DNN, for training in the second stage. After the two-stage training, given a testing instance, the testing prediction is calculated through the entire model by the fitted forest and DNN. Note that for implementation purpose, the forest prediction feature **f**_*i*_, ∀*i* is one-hot encoded as shown in Fig. 1. This is the same operation as with the label vectors **y**_*i*_, since the final output dimension from DNN is two. Consequently, the actual input for the DNN in our implementation is an *n* × *M* × 2 tensor rather than an *n* × *M* matrix **F**.

For the DNN model, the activation functions are the rectified linear unit (ReLU)^22^ with the form (in scalar case)

$${\sigma }_{ReLU}(x)=max(x,\mathrm{0).}$$

This activation has an advantage over sigmoid and hyperbolic tangent as it can avoid the vanishing gradient problem^23^ during optimization. For the optimization algorithm, We choose the Adam optimizer^24^ as it is the most widely used variant of traditional gradient descent algorithms in deep learning nowadays. Also, we use the mini-batch training strategy by which the optimizer randomly trains a small proportion of the samples in each iteration. Details about the Adam optimizer and mini-batch training can be seen in deep learning literature^21,24^.

The classification performance of the fDNN model is associated with both hyper-parameters for the forest and for the DNN. Forest hyper-parameters include number of trees in the forest, and tree related parameters such as tree depth, minimum splitting sample size etc. DNN hyper-parameters are architecture related parameters such as the number of layers and the number of hidden neurons in each layer, regularization related parameters such as the dropout proportion and the penalty scale of regularizers, model training related parameters such as the learning rate and the batch size. Those hyper-parameters can be fine-tuned using advanced hyper-parameter optimizing algorithm such as Bayesian Optimization^25^. However, in this work, our primary interest is to examine the performance of fDNN compared to ordinary classifiers under same or similar settings, instead of fine tuning a “best-of-all” model for specific datasets. Therefore, the hyper-parameters are simply chosen by convention or tuned using grid search with synthetic validation datasets in a feasible hyper-parameter space. The method is implemented in Python with packages Scikit-learn^26^ and Tensorflow^27^.

### Synthetic data generation

The goal of simulation experiments is to mimic disease outcome classification using gene expression data, where $n\ll p$ and effective features are extremely sparse and correlated, and explore the performance of our new model compared to ordinary classification methods. We compare our fDNN method with usual random forests and DNN, which account for the two parts of fDNN respectively. Through the numerical experiments, we are intended to show that fDNN is able to improve the classification performance of pure random forests or DNN, and the better performance cannot be achieved simply by increasing the complexities of the two ordinary classifiers. Robustness is also tested as we simulate datasets that do not fully satisfy the correlated feature assumption, and apply the new method to examine whether it can still achieve a reasonable performance.

For a given number of features *p*, we first generate a latent feature network using the preferential attachment algorithm^28^. Each node of the network represents one feature. The resulting network is scale-free with a power-law degree distribution. That means only a few features in this network have relatively large number of “neighbors”. Defining the distance between two features in the network as the shortest path between them, a *p* × *p* distance matrix *D* recording pairwise distances among features is then calculated. Next, the distance matrix is transformed into a covariance matrix Σ by letting

$${{\rm{\Sigma }}}_{ij}={0.7}^{{D}_{ij}},i,j=1,\ldots ,p.$$

Here by convention the diagonal elements of *D* are all zeros meaning the distance between a feature to itself is zero, and thus the diagonal elements of Σ are all ones. Since only a few features have high connections in the feature network, this fact is reflected in the Σ matrix. Utilizing Σ as the covariance matrix for generating sample instances, we are then able to achieve the goal that features have sparse and correlated structures. *n* multivariate Gaussian samples are simulated forming the data matrix **X** = (**x**_1_, …, **x**_*n*_)^*T*^ i.e.

$${{\bf{x}}}_{i} \sim {\mathscr{N}}({\bf{0}},{\rm{\Sigma }}),i=1,\ldots \,n,$$

where $n\ll p$ for imitating real gene expression situations.

To generate outcome variables, we select a subset of all features to be the “true” predictors. The selection is conducted as follows: in the generated feature network mentioned above, we randomly select part of the high-degree features as “cores”, and a portion of their neighbors are also randomly selected. In this way, the true predictors satisfy: (1) sparsity, since only a few of all are high-degree features and only part of the neighbors are selected. (2) correlated structure, since the “core” features have much higher correlation with their neighbors than other distant features. Denoting the number of true predictors as *p*_0_, we sample a set of parameters ${\boldsymbol{\beta }}=({\beta }_{1},\ldots ,{\beta }_{{p}_{0}}{)}^{T}$ and an intercept *β*_0_ within a certain range. In our experiments, we first sample *β*’s from the interval (0.05, 0.1), and some of the parameters are randomly turned into negative, so that we accommodate both positive and negative coefficients. Finally, the outcome variable ***y*** is generated through a logistic regression model

$$\begin{array}{rcl}Pr({y}_{i}\mathrm{=1|}{{\bf{x}}}_{{\bf{i}}}) & = & logi{t}^{-1}({{{\bf{x}}}_{{\bf{i}}}}^{T}\beta +{\beta }_{0})\\ {y}_{i} & = & {\mathscr{J}}(Pr({y}_{i}=\mathrm{1|}{{\bf{x}}}_{{\bf{i}}}) > t),\,i=1,\ldots \,\,n,\end{array}$$

where ${\mathcal L}$(·) is the indicator function, *t* is a threshold and *logit*(⋅) is the logit function

$$logit(x)=log\,(\frac{x}{1-x}).$$

The inverse *logit*^−1^ is equivalent to a binary class softmax function.

Following the above procedure, we simulate a set of synthetic datasets with 5,000 features and 400 samples. Since we are considering cases with extremely low signal-to-noise ratio, we examine different numbers, i.e. 10, 20, 30 40, and 50 of true predictors, corresponding to 1–5 cores among all the high-degree features. Also, in reality, the true predictors may not be only distributed at the high-degree nodes and their neighbors in the latent feature network. Instead, a few of the true predictors can be quite scattered. To test model robustness in this possible circumstance, in addition to generating datasets following the above procedure, we also simulate another series of datasets where 50% of the true predictors are randomly selected among the entire feature network rather than from high-degree features and their neighbors. We call these two sets of data “clustered” case and “scattered” case respectively, according to the property of predictor structures.

### Real datasets

We apply the fDNN method to two real datasets. The first is the single-cell RNA-seq data on bone marrow cells^29^ (GSE99095). The dataset consists of a gene expression matrix with 17,258 genes in 391 control cells from healthy donors, and 588 cells from 5 patients with bone marrow failure. The original study has found the cell populations are diverse both in patients and in healthy donors, with patient cells showing higher diversity due to the existence of aneuploid cells^29^. We obtain the normalized expression matrix from the Gene Expression Omnibus (GEO). Our interest is to test the method’s capability to classify the source of the cells, i.e. healthy/diseased, despite the presence of cell diversity within each class.

The other dataset we study is GSE106291, which contains the RNA-seq expression profiles of 23,368 genes from 250 acute myeloid leukemia patients under intensive treatment^30^. The primary clinical outcome is treatment resistance. Each patient was labeled as either resistant or sensitive to the treatment. We aim at classifying the two responses with the gene expression data. From the original normalized expression matrix, we delete genes with more than 10% zero measurements, resulting in the final data matrix with 11,068 features and 250 columns. For each feature, the expression value is Z-score transformed, i.e. the expression value minus the mean across all patients and then divided by the standard deviation.

The two datasets are suitable for testing our method, because the datasets fall into the $n\ll p$ category, the features are correlated due to their functional relations, and only a small portion of the features are expected to contribute to the biological mechanism that generated the class membership.

## Results and Discussion

### Simulation results

In our simulation studies, the fDNN had 300 trees in the forest part and three hidden layers in the DNN part, with 256, 64 and 16 hidden neurons respectively. Note the 300-tree forest served as a supervised feature detector for the downstream DNN, and the feature space was shrunk from *p* = 5000 to *M* = 300, mediating intractable $n\ll p$ situation since *n* and the input dimension *M* are now at the same magnitude. We also observed that adding more hidden layers to DNN resulted in similar prediction performance, hence the three-hidden layer architecture was finalized as a parsimonious choice. To compare, we also recorded the prediction performance from the 300-tree forests (RF_300) in fDNN and experimented with a DNN classifier (DNN_3_256) with the same architecture as the one in fDNN. Moreover, we tested additional random forests with 500 trees (RF_500) and DNN with one more hidden layer (1024 neurons) at the top (DNN_4_1024), for the reason mentioned in the Methods section. For each of the data generation settings, 10 datasets were generated, and all methods mentioned above were applied on the data. For each simulated dataset, we randomly split the dataset into training and testing sets at a 4:1 ratio. The final testing classification performances were then averaged across the ten datasets. All the classification results were evaluated by the area under the receiver operating characteristic (ROC) curve (AUC).

**Figure 2:**
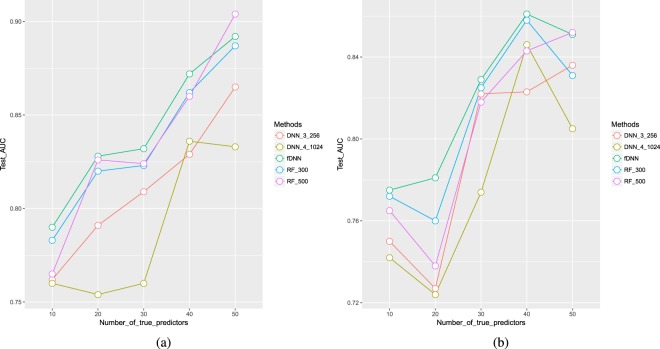
Plots of the classification comparison in Table 1. Cases: (**a**) clustered (**b**) scattered.

Table 1 and Fig. 2 show the results of the simulation experiments. Corresponding to the “clustered” case, Fig. 2(a) shows the fDNN method outperformed RF_300 and DNN methods, and performed better than RF_500 in most cases. As the number of true predictors increased, there were increasing trends for all of the methods, with a few exceptions due to the randomness of data generation. The trends for fDNN and RF_300 are quite parallel. However, the downstream DNN in fDNN always improved the prediction from the forests. Note that DNN_4_1024 was actually worse than DNN_3_256, and this makes sense because under $n\ll p$ the deeper neural network is more affected by the pitfall of the overfitting phenomenon. Hence, it in turn demonstrated the necessity for constructing a model with reduced feature dimension as in fDNN to get around this issue.

**Table 1:** Classification comparison of the forest Deep Neural Network (fDNN) method, deep neural networks (DNN) and random forests (RF).

Case	Clustered					Scattered				
# true predictors	10	20	30	40	50	10	20	30	40	50
fDNN	0.79	0.828	0.832	0.872	0.892	0.775	0.781	0.829	0.861	0.851
DNN_3_256	0.762	0.791	0.809	0.829	0.865	0.75	0.727	0.822	0.823	0.836
DNN_4_1024	0.76	0.754	0.76	0.836	0.833	0.742	0.724	0.774	0.846	0.805
RF_300	0.783	0.82	0.823	0.862	0.887	0.772	0.76	0.825	0.858	0.831
RF_500	0.765	0.826	0.824	0.86	0.904	0.765	0.738	0.818	0.843	0.852

Statistics are the classification accuracies measured by AUC.

As for the “scattered” case (Fig. 2(b)), fDNN was still the best among the five, while overall AUC’s slightly decreased compared to Fig. 2(a). This is because for neural network methods, DNN inherently tackles correlated features. When the correlation among features decreased, the performance of DNN_3_256 and DNN_4_1024 in (b) became worse than that in Fig. 2(a). At the same time, although not as directly affected as DNN methods, decreased feature correlation also deteriorated the performance of RF_300 and RF_500. Recall we only selected a proportion of high-degree feature neighbors as the true predictors. The remaining neighbors could also be informative when constructing decision trees, due to their high correlation with the true predictors. Consequently, compared to the scattered case where half of the selected true predictors could hardly be connected by others in the feature network, the clustered case is easier for random forests as the chance of selecting “relevant” predictors is higher.

In summary, the simulation experiments demonstrated that our newly proposed fDNN classifier had better classification performance compared to ordinary random forests or deep neural networks alone, in the situation that $n\ll p$ and signals are sparse and correlated. Moreover, the improved performance could not be achieved by simply increasing the model complexities for random forests and DNN. The method was also robust as it outperformed other methods in both of the clustered and scattered cases.

### Real data results

**Figure 3:**
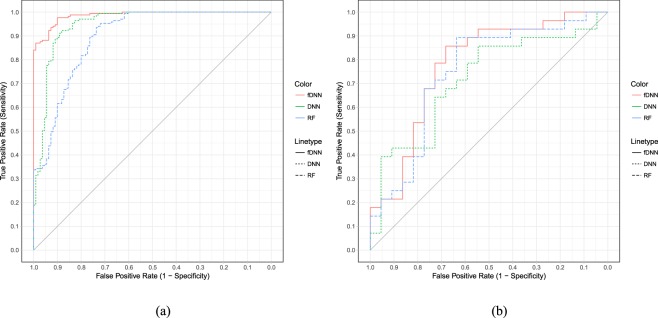
ROC plots for (**a**) GSE99095 and (**b**) GSE106291.

For each of the two datasets, we again randomly divided all samples into training and testing sets. For GSE99095, we had 700 training and 279 testing samples; for GSE106291, the numbers were 200 and 50 respectively. The three classifiers, fDNN, DNN, and RF were trained on the training samples. Hyper-parameters were chosen by cross-validation using the training datasets. The classification performance was again evaluated by the testing AUC of ROC. The computation times of fDNN training plus testing were 69.9 seconds for GSE99095 and 40.8 seconds for GSE106291 respectively, on a workstation with dual Xeon Gold 6136 processors, 192 GB RAM, and a single Nvidia Quadro P5000 GPU. Tables 2 and 3 list the detailed architectures for each classification methods and summarize the testing results for GSE90995 and GSE106291 respectively. Corresponding ROC plots are shown in Fig. 3(a,b).

**Table 2:** Testing results for the GSE99095 dataset.

Method	Architecture	Testing AUC
fDNN	400Trees + 256 + 64 + 16	**0.986**
DNN	1024 + 512 + 128 + 16	0.949
RF	1000Trees	0.897

Numbers in the architecture column denote the number of trees in Random Forest and the number of hidden neurons in neural network methods.

**Table 3:** Testing results for the GSE106291 dataset.

Method	Architecture	Testing AUC
fDNN	500Trees + 256 + 64 + 16	**0.778**
DNN	1024 + 256 + 16	0.751
RF	1000Trees	0.716

Numbers in the architecture column denote the number of trees in Random Forest and the number of hidden neurons in neural network methods.

From the tables and the ROC plots, we see that fDNN was able to obtain better classification results in terms of ROC, compared to traditional DNN and RF classifiers. All three methods performed reasonably well on GSE99095, which contained close to 1000 samples. Although RF performed better than DNN in simulations, in the real dataset DNN achieved slightly better testing results. Our fDNN method, by learning sparse representation using RF as a feature detector, improved over the two methods in terms of testing data classification. GSE106291 had a smaller sample size of 250, which tested the limits of the methods. The small sample size may be the reason why all three methods performed worse. Still the fDNN achieved slightly better testing data classification error rate, indicating its applicability on gene expression datasets with relatively small sample size.

In real analysis of gene expression data, one may not only be concerned about the prediction results, but also be interested in features with major contribution to the classification, as those significant genes can reveal biological mechanisms. After fitting the fDNN model, we employed a newly developed variable ranking mechanism, which combined the variable importance calculation in ordinary random forests and the Connection Weights (CW) method^31^ introduced in neural networks, to calculate a score for each gene as the variable importance in fDNN.

In random forests, variable importance is quantified by cumulating the decrease of impurity caused by splitting at a certain feature across all the trees. Based on this fact, the forest ${\mathcal F}$ in fDNN is also able to record feature importance during fitting. Moreover, importance scores of features in each tree ${\mathscr{J}}$ are also available, resulting in a *p* × *M* tree importance matrix **S**, where again *p* is the number of features and *M* is the number of trees in ${\mathcal F}$.

For an ordinary DNN, the CW method tries to quantify the contribution of an input variable by summing over all the absolute values of the weights connecting the variable and the first hidden layers, assuming all input data are standardized. Mathematically, we have

$${u}_{j}=\sum _{k=1}^{p}\,|{w}_{jk}^{(in)}|,$$

where *u*_*j*_ is the importance score for feature *j*, *w*^(*in*)^ denotes weights between the input and first hidden layers. The same logic applies in fDNN, but instead of calculating the importance of each feature, the CW method helps compute the importance of each tree output by

$${v}_{j}=\sum _{k=1}^{M}\,|{w}_{jk}^{(in)}|,$$

and here *v*_*j*_ is the importance score for ${{\mathscr{J}}}_{j}$. Now we have both quantified the feature importance in the forest part and the tree importance in the DNN part. Denoting ${{\bf{v}}}^{\ast }={({v}_{1}^{\ast },\ldots {v}_{M}^{\ast })}^{T}$ as the normalized importance scores for all the trees in DNN with ${\sum }_{i=1}^{M}\,{v}_{i}^{\ast }=1$, we finally combine the two parts of fDNN with

$${\rm{\lambda }}={\bf{S}}{{\bf{v}}}^{\ast },$$

where ***λ*** = (*λ*_1_, … *λ*_*p*_)^*T*^ is the final importance of the original features.

Applying this feature evaluation procedure to our real data examples, we obtained a ranked gene importance lists for GSE99095 and GSE106291 respectively. For GSE99095, we analyzed top 1% ranked genes from both fDNN and RF for comparison purpose. The reason for comparing RF is that it is commonly used as a variable importance ranking tool. Among the top 1% (172) genes selected by the two methods, 52 genes overlap. GO enrichment results are shown in Table 4.

**Table 4:** The top 10 overrepresented GO biological processes by the top 1% genes selected in fDNN from GSE99095 data, after manual removal of redundant GO terms.

GOBPID	Pvalue	Term	Significant in RF selected genes (p < 0.01)	Significant in genes uniquely selected by fDNN (p < 0.01)
GO:0070125	0.000319438	mitochondrial translational elongation		Y
GO:1990542	0.000319438	mitochondrial transmembrane transport		Y
GO:0006119	0.000431138	oxidative phosphorylation	Y	Y
GO:0006412	0.000524598	translation		Y
GO:0048534	0.000553723	hematopoietic or lymphoid organ development	Y	
GO:0007229	0.00166512	integrin-mediated signaling pathway	Y	
GO:0098754	0.00166512	detoxification	Y	
GO:0016073	0.002434088	snRNA metabolic process		
GO:0007599	0.004203111	hemostasis		
GO:1903018	0.00560232	regulation of glycoprotein metabolic process		

Among the top 10 fDNN selected pathways, one of the major themes is related to the mitochondria. The top three biological processes include the synthesis of mitochondrial proteins, mitochondrial transport, and the process of energy generation through oxidative phosphorylation. The results indicate that at the cellular level, mitochondria biogenesis and energy production is associated with the bone marrow failure outcome. Comparatively, RF also identified the oxidative phosphorylation as a significant process, but not the mitochondrial protein biosynthesis and transport processes. Both fDNN and RF found the hematopoietic process and the integrin pathway, which are integral parts of blood cell development. The fDNN selected the hemostasis pathway, which is another important part of blood cell regulations.

For the GSE106291 data, as the total number of genes under study is smaller, to maintain sufficient statistical power in gene set enrichment analysis, we compared the top 2% most important genes from fDNN and RF. Between the two lists of top 221 genes, 81 genes overlap. As shown in the enrichment analysis results (Table 5), both fDNN and RF selected chemotaxis as the top GO term. However, the second term selected by fDNN, “myeloid leukocyte activation”, was not found by RF. Given the nature of the disease, and the clinical response under study, i.e. treatment resistance, it is expected that the myeloid activation pathways play a critical role. The fDNN also selected small molecule metabolism, exocytosis, and cAMP response as important processes in the resistance to the treatment, which have been implicated in chemotherapy response in other types of cancer. Overall, fDNN selected different important genes from RF, and the biological functions overrepresented by the fDNN selected genes are plausible.

**Table 5:** The top 10 overrepresented GO biological processes by the top 2% genes selected in fDNN from GSE106291 data, after manual removal of redundant GO terms.

GOBPID	Pvalue	Term	Significant in RF selected genes (p < 0.01)	Significant in genes uniquely selected by fDNN (p < 0.01)
GO:0006935	0.000640609	chemotaxis	Y	
GO:0002274	0.001091917	myeloid leukocyte activation		Y
GO:0062014	0.001389434	negative regulation of small molecule metabolic process		Y
GO:0016477	0.001567641	cell migration		Y
GO:0045055	0.002003684	regulated exocytosis		Y
GO:0060078	0.002129227	regulation of postsynaptic membrane potential		
GO:0030334	0.00244581	regulation of cell migration		Y
GO:0030501	0.002766529	positive regulation of bone mineralization		
GO:0061045	0.002925766	negative regulation of wound healing		
GO:0071320	0.003321428	cellular response to cAMP		Y

## Conclusion

We presented a new forest - deep neural network classifier aimed at $n\ll p$ classification problems for clinical outcome prediction using gene expression data. Its machinery relies on supervised learning feature representations from a forest and training classifiers in a deep neural network. Simulation experiments have shown its relatively higher classification accuracy compared to existing methods, and the real data application demonstrated the utility of the new model.
